# Acute Effect of Caffeine on Cerebral Activity in Older Individuals with and without Alzheimer's Disease using Event‐Related Spectral Perturbation

**DOI:** 10.1002/alz70858_097518

**Published:** 2025-12-24

**Authors:** Britman Salcedo Pumaccola, Saulo J. Gil, Danilo J. Calhes, Leonardo Gonçalves Ferreira, Thays Martins Vital Silva, Thiago Emmanuel Medeiros, Iane de Paiva Novais, Angelica Miki Stein, Francisco J. Fraga

**Affiliations:** ^1^ Federal University of ABC (UFABC), Santo André, SP, Brazil; ^2^ Technological Federal University of Paraná (UTFPR), Curitiba, PR, Brazil; ^3^ UNESP ‐ Universidade Estadual Paulista, Biosciences Institute, Rio Claro, SP, Brazil

## Abstract

**Background:**

Alzheimer's disease (AD) is a neurodegenerative disorder characterized by progressive cognitive decline. Caffeine has been linked to cognitive enhancements and a reduced risk of developing AD, potentially due to adenosine receptor inhibition and increased neuronal activity. However, its effects on cerebral activity, particularly in individuals with AD, remain unclear. This study investigates the impact due to caffeine ingestion on cerebral activity using Event‐Related Spectral Perturbation (ERSP) analysis of EEG signals in older adults with and without AD.

**Methods:**

The study was conducted with 12 older adults: 5 with AD and 7 healthy controls (CTRL). Subjects from both groups were matched for sex, age, and education. Participants underwent two experimental sessions, receiving either a caffeine (5 mg/kg) or a placebo capsule, followed by EEG recordings at baseline and 50 minutes post‐ingestion. The Oddball paradigm was employed to elicit event‐related potentials. Both the frequent (not significant) stimuli and the target (significant) were analyzed. EEG signals were evaluated using time‐frequency methods to quantify ERSP changes. Statistical analysis tests were performed by the non‐parametric Montecarlo method, with Cluster correction for multiple comparisons. EEGLAB was used both for pre‐processing (filtering and artifact removal) as well as for the ERSP and statistical analyses.

**Results:**

For the frequent stimuli, caffeine intake displayed significant differences at all electrodes in the AD group, whereas in the CTRL group significant differences were observed only at frontal and occipital electrodes (*p* < 0.05). Frequency bands were also different between groups: while AD presented significant differences in all EEG bands, CTRL group differences were concentrated on the gamma band, except the F8 electrode, which also presented differences in delta, theta, and alpha bands (*p* < 0.05). Regarding the target stimuli, CTRL showed significant differences in all EEG bands and in the areas frontal, central, parietal, and occipital, while AD exhibited differences in bands theta, alpha, beta, and gamma, at frontal, temporal, parietal, and occipital electrodes (*p* < 0.05).

**Conclusions:**

Caffeine differentially modulates cerebral activity in older adults with and without AD. Expanding the sample size could provide a more comprehensive understanding of caffeine's role in modulating cerebral activity and its implications for AD treatment strategies.

